# A Second Sarah

**Published:** 1890-01

**Authors:** R. A. Taylor

**Affiliations:** Nevada, Texas


### A SECOND SARAH.

NEVADA, TEXAS, January 6, 1890.

*Editor Daniel's Texas Medical Journal:*

A few days since I delivered a lady of a healthy female child after an easy labor, the father being sixty-four and the mother fifty-four years of age. The lady had not been pregnant for eleven years. Is not this an unusual age for child bearing?

The mother blames my medicine, but I contend if she had taken nothing but my medicine it would not have been thus with her.

Yours truly,

R. A. TAYLOR.

